# Supplementary material for: Premature termination codon readthrough in Drosophila varies in a developmental and tissue-specific manner
Source: Sci Rep. 2020 May 22;10:8485. doi: 10.1038/s41598-020-65348-8 (PMC7244557; doi:10.1038/s41598-020-65348-8)
Supplement: Supplementary file 2 — Supplementary Figures [file 41598_2020_65348_MOESM2_ESM.pdf]

Title: Premature termination codon readthrough in *Drosophila* varies in a developmental and tissue-specific manner

Authors: Yanan Chen[1, 2], Tianhui Sun[2], Zhuo Bi[1, 2], Jian-Quan Ni[3], Jose C. Pastor-Pareja[2, 4*] and Babak Javid[1, 5*]

Affiliations:
[1] Center for Global Health and Infectious Disease Research, Collaborative Innovation Center for the Diagnosis and Treatment of Infectious Diseases, Tsinghua University School of Medicine, Beijing, China 100084
[2] School of Life Sciences, Tsinghua University, Beijing, China 100084
[3] Tsinghua University School of Medicine, Beijing, China 100084
[4] Tsinghua-Peking Joint Center for Life Sciences, Tsinghua University, Beijing, China 100084
[5] Beijing Advanced Innovation Center in Structural Biology

Supplementary Figures

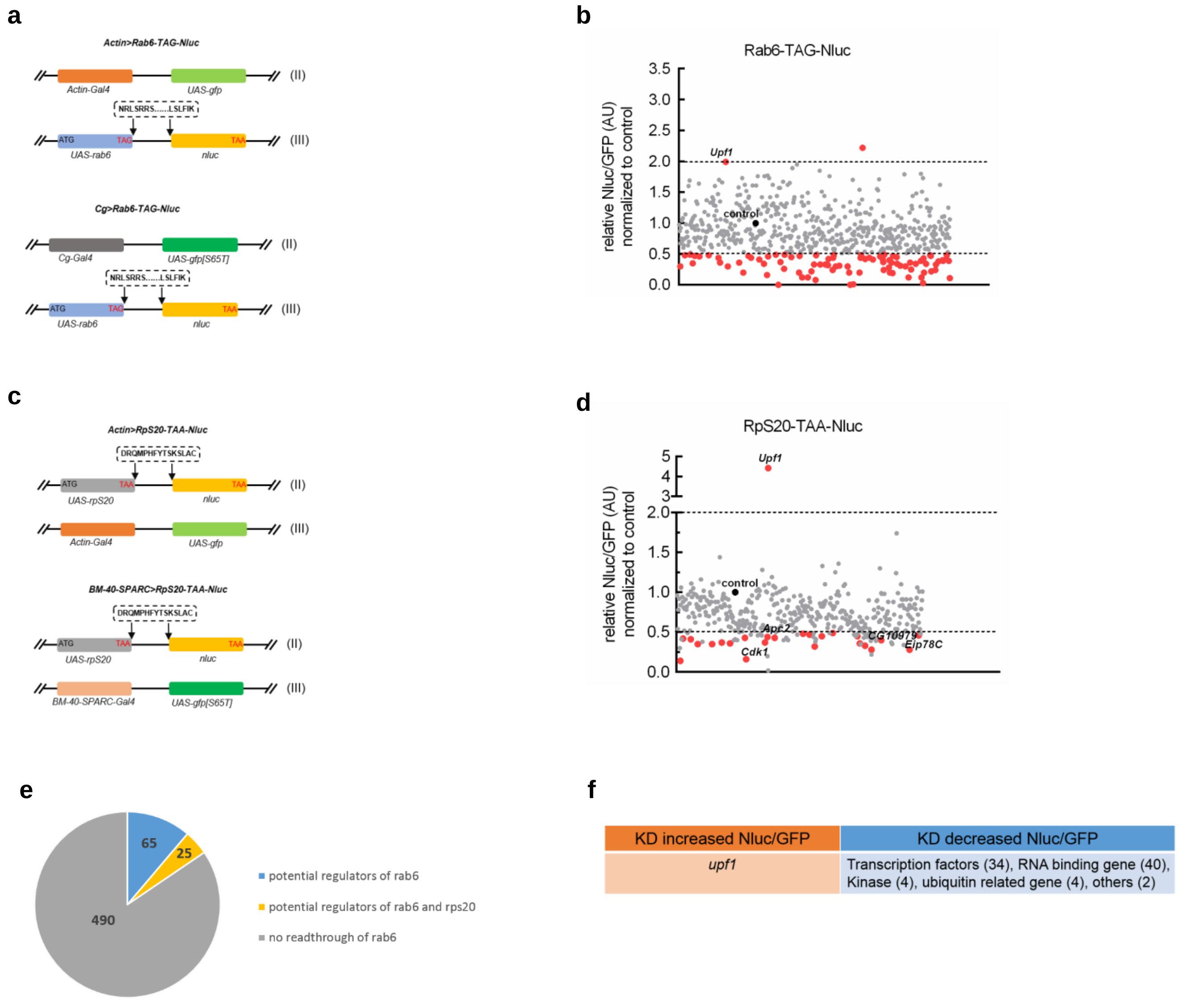

Supplementary figure 1. A candidate forward genetic screen identifies regulators of *rab6* readthrough regulation.
a, Schematic of reporter fly line used in the primary forward genetic screen. The reporter fly line was crossed with a set of *UAS-RNAi* flies to knock-down target genes. **b**, Result of the candidate forward genetic screen identifying potential regulators of translational readthrough of *rab6*. The control (black dot) represents relative readthrough rate of the progeny of the reporter fly line (♀) and *w*^{1118} (♂) and normalized to 1. Hits (above or below dotted lines) were defined as relative readthrough rates 2x higher or lower than control. **c**, Schematic of reporter fly line used in the secondary forward genetic screen. **d**, Result of the candidate forward genetic screen identifying potential regulators of translational readthrough of *rps20*. Hits of *rab6* were re-tested by rps20-based reporter, and red dots represent hits that showed the same result in both reporter fly lines. Results are summarised by the pie chart in **(e)**. **f)** *upf1* participates in both *rab6* and *rps20* readthrough regulation.

a

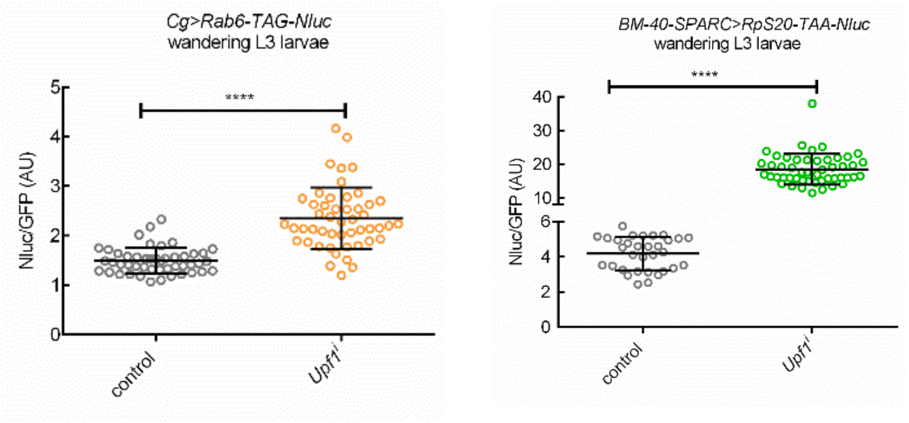

b

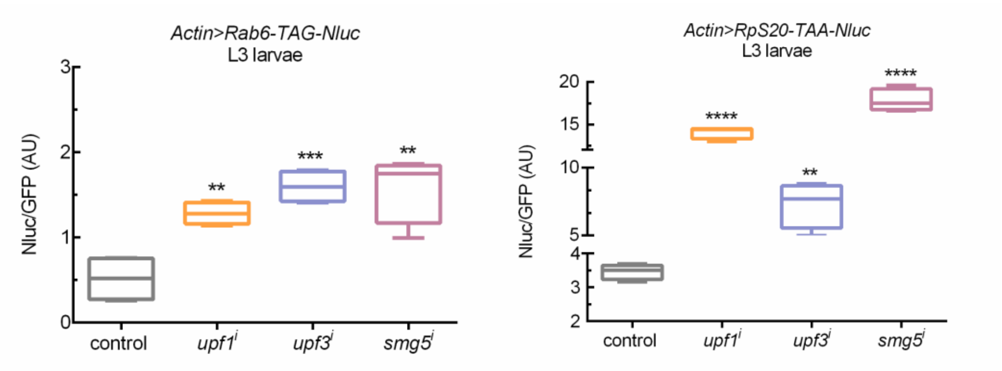

Supplementary figure 2. Readthrough rates of reporter flies are sensitive to NMD.

a, Knock-down of *upf1* results in increased readthrough products of both *rab6* and *rps20*. Each single wandering L3 larva was collected and readthrough level was independently calculated by measuring Nluc activity and dividing by GFP to control for variation in UAS-driven protein expression. Means between control and *upf1* knockdown groups were compared by two-tailed, non-parametric Mann-Whitney tests (data did not pass normality tests). **b**, Knock-down of NMD associated factors results in increased readthrough products of both *rab6* and *rps20*. *upf1* (BDSC_43144), *upf3* (BDSC_58181) and *smg5* (BDSC_62261) were knocked down efficiently by Actin promoter. Data represent means of three independent experiments $\pm$ s.d. in C. ***P* < 0.01, ****P* < 0.001, *****P* < 0.0001 by Student's t-test.

.

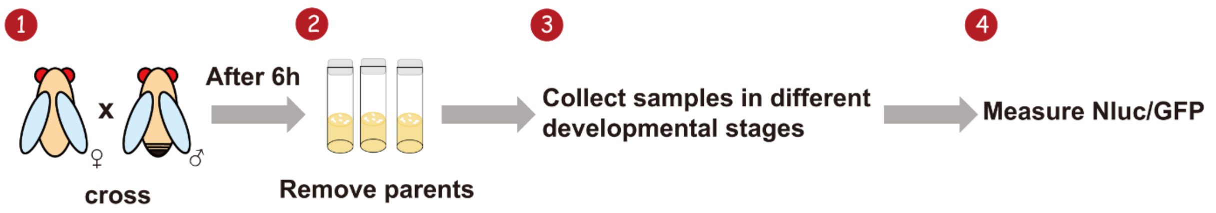

Supplementary figure 3. Schematic showing how relative readthrough rates were measured over development stages.
Parent transgenic fly lines *UAS-transgene-STOP-Nluc* and *Actin-Gal4* were crossed to generate the progeny *Actin>transgene-STOP-Nluc*. 6 hours following the cross, parents were removed to ensure progeny were all in a similar developmental stage. Readthrough rates were measured in cell-lysates of whole flies.

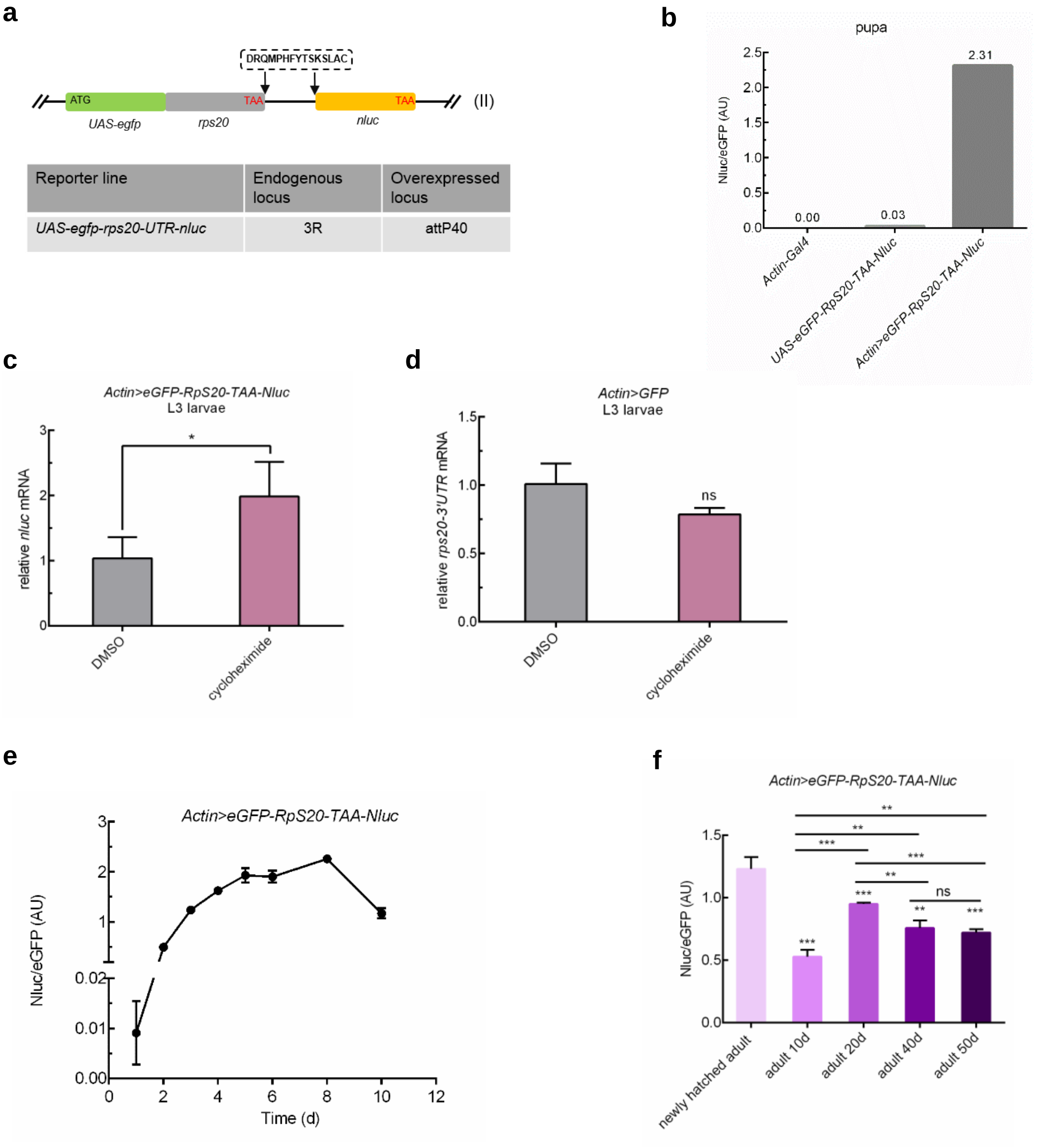

Supplementary figure 4. PTC readthrough of *rps20* is also regulated in a developmental stage-dependent manner.

a, Schematic for construction of the in-frame stop codon readthrough reporter. Here, the peptide sequence DRQMPHFYTSKSLAC were encoded by extension 3'UTR of *rps20*. **b**, As for the reporter measuring readthrough of *rab6* (see Figure 3B), Nluc was only detected when both the reporter and Gal4 were expressed. **c, d)** The reporter transcript of *rps20* is subjected to NMD. Reporter transcript abundance was assessed by real-time RT-PCR with *nluc* primer **(c)**. Endogenous *rps20* mRNA abundance was measured by real-time RT-PCR with *rps20-3'UTR* primer **(d)**. *rp49* abundance was used for normalization. **e**, Readthrough of *rps20* varied during fly life-cycle. **f**, Higher readthrough level in old flies is not aging associated.

Data represent means of three independent experiments ± s.d. **P* < 0.05, ***P* < 0.01, ****P* < 0.001; ns, *P* > 0.05 by Student's t-test.

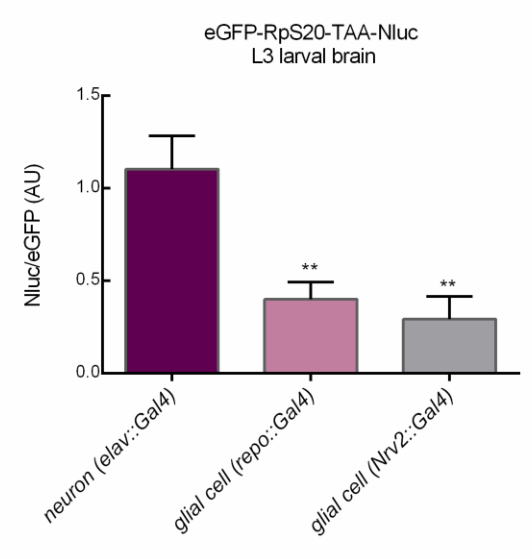

Supplementary figure 5. PTC readthrough of *rps20* is higher in neurons

Drosophila larval neurons undergo significantly higher readthrough level than glial cells. L3 larval brain were dissected. *rp49* abundance was used for normalization.

Data represent means of three independent experiments ± s.d. ***P* < 0.01 by Student's t-test.

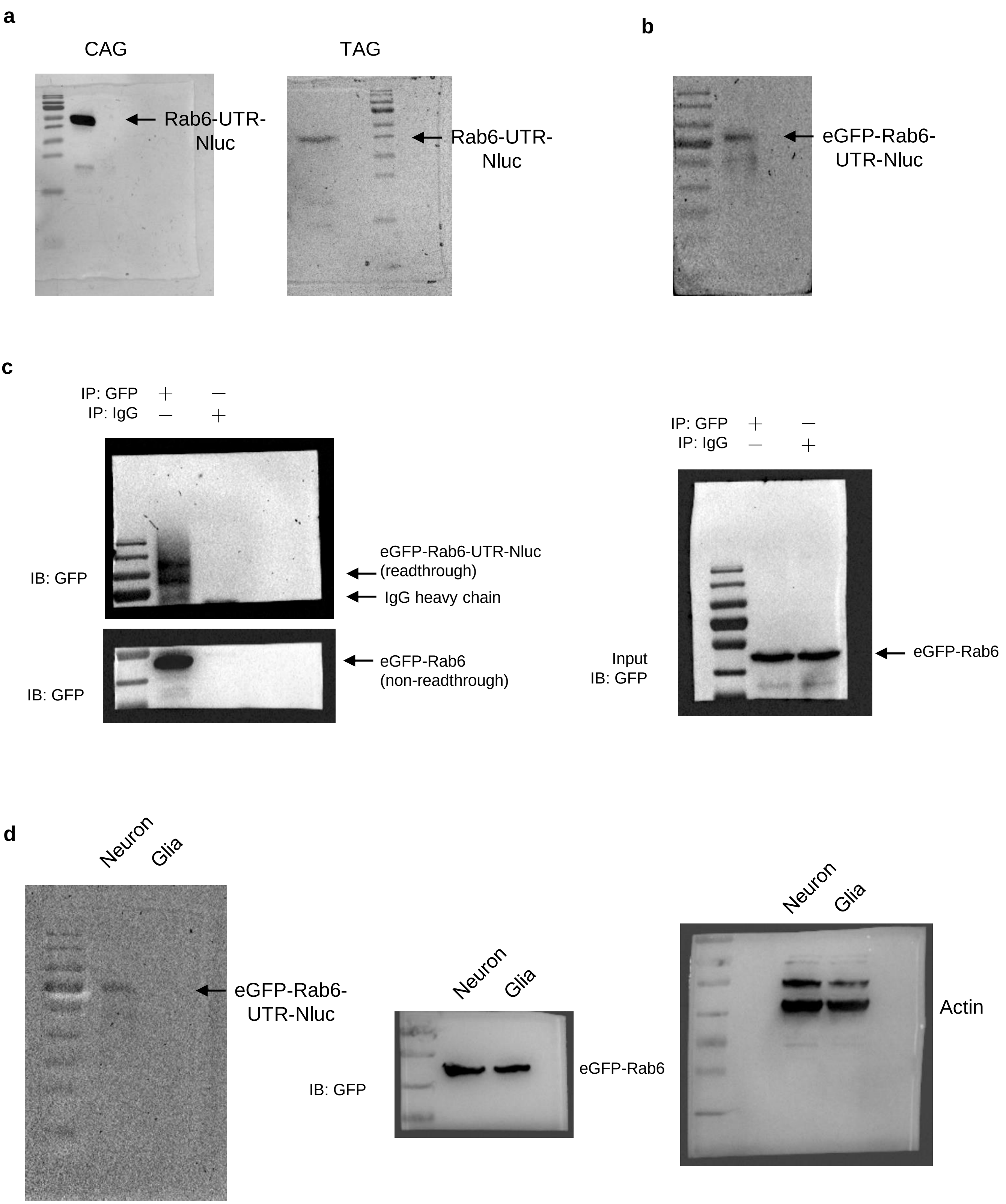

Supplementary figure 6. Full length gels and blots

a, Full length gels of Fig. 1f. **b**, Full length gel of Fig. 3c. **c**, Full length blots of Fig.3d. **d**, Full length gel and blots of Fig.4g.
